# Supplementary figures and images for: Protein expression of prenyltransferase subunits in postmortem schizophrenia dorsolateral prefrontal cortex
Source: Transl Psychiatry. 2020 Jan 10;10:3. doi: 10.1038/s41398-019-0610-7 (PMC7026430; doi:10.1038/s41398-019-0610-7)

Supplementary Figure S1

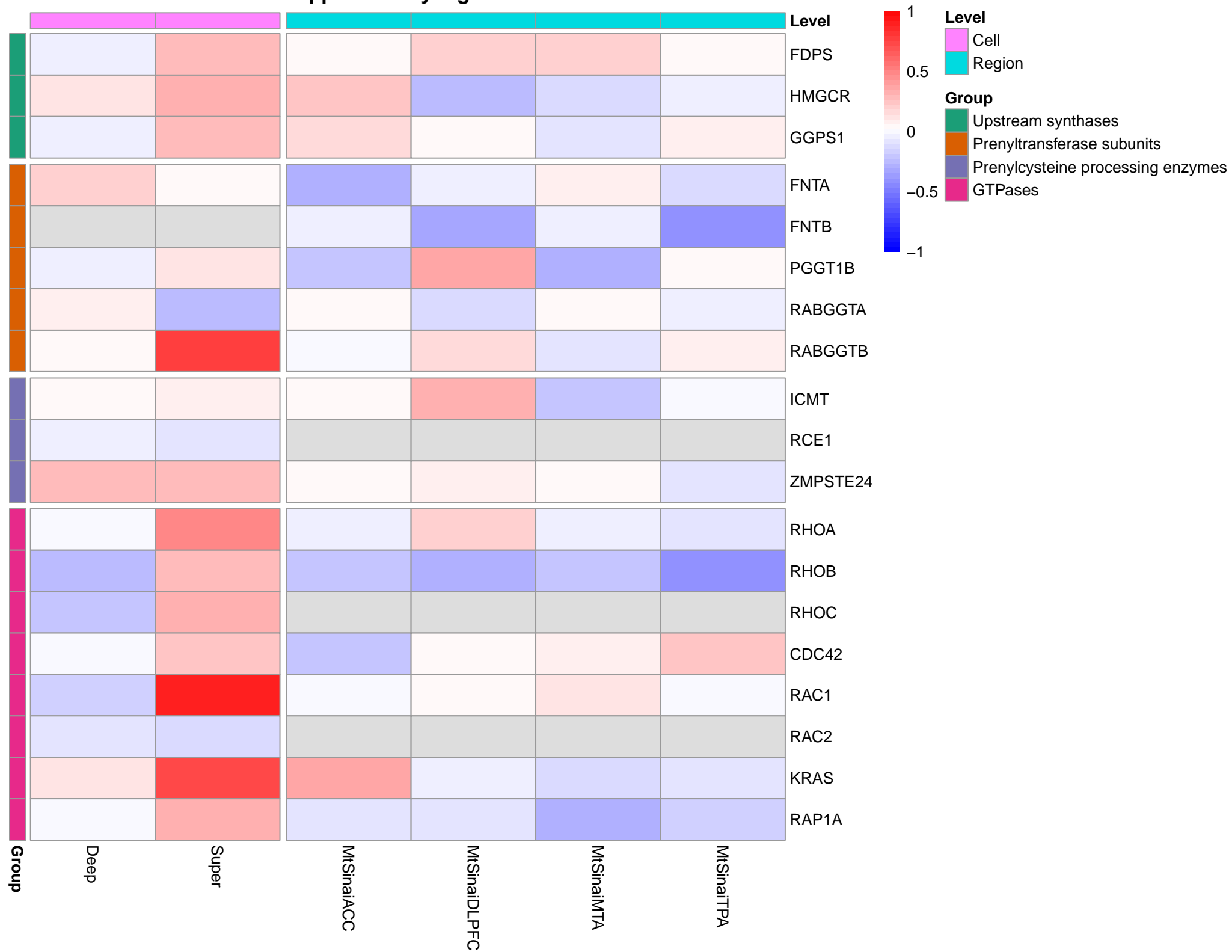

Supplement: Supplementary file 2 — Supplementary Figure S1 [file 41398_2019_610_MOESM2_ESM.pdf]
