# Supplementary material for: Protein expression of prenyltransferase subunits in postmortem schizophrenia dorsolateral prefrontal cortex
Source: Transl Psychiatry. 2020 Jan 10;10:3. doi: 10.1038/s41398-019-0610-7 (PMC7026430; doi:10.1038/s41398-019-0610-7)
Supplement: Supplementary file 3 — Supplementary Table S1 [file 41398_2019_610_MOESM3_ESM.pdf]

Supplementary Table S1. Expression differences of prenylation-associated genes from transcriptomic datasets

		MtSinaiMTA			MtSinaiTPA			MtSinaiACC			MtSinaiDLPFC			DLPFC_Super			DLPFC_Deep		
	**Gene Symbol**	**FC**	**LogFC**	**p-value**	**FC**	**LogFC**	**p-value**	**FC**	**LogFC**	**p-value**	**FC**	**LogFC**	**p-value**	**FC**	**LogFC**	**p-value**	**FC**	**LogFC**	**p-value**
	**FDPS**	**1.15**	**0.20**	0.05	1.02	0.02	0.81	1.04	0.06	0.62	**1.16**	**0.21**	0.28	**1.22**	**0.28**	0.19	-1.02	-0.03	0.74
**Upstream synthases**	**HMGCR**	**-1.10**	**-0.14**	0.34	-1.04	-0.05	0.81	**1.19**	**0.26**	0.23	**-1.18**	**-0.24**	0.15	**1.24**	**0.31**	0.22	1.09	**0.13**	0.45
	**GGPS1**	-1.06	-0.09	0.54	1.06	0.09	0.44	**1.14**	**0.18**	0.13	1.03	0.05	0.66	**1.21**	**0.28**	0.19	-1.04	-0.05	0.60
	**FNTA**	1.06	0.08	0.67	-1.08	-0.11	0.25	**-1.21**	**-0.27**	0.07	-1.01	-0.02	0.90	1.04	0.05	0.74	**1.17**	**0.22**	0.06
	**FNTB**	-1.03	-0.04	0.87	**-1.34**	**-0.42**	**0.004**	-1.03	-0.05	0.77	**-1.25**	**-0.32**	0.08						
**Prenyltransferase subunits**	**PGGT1B**	**-1.21**	**-0.28**	0.09	1.03	0.04	0.79	**-1.15**	**-0.20**	0.33	**1.31**	**0.39**	0.21	**1.10**	**0.14**	0.49	-1.04	-0.06	0.55
	**RABGGTA**	1.03	0.04	0.73	-1.03	-0.05	0.71	1.02	0.02	0.88	**-1.10**	**-0.14**	0.19	**-1.17**	**-0.23**	0.05	1.05	0.07	0.47
	**RABGGTB**	-1.06	-0.09	0.50	1.05	0.06	0.57	-1.00	-0.01	0.96	**1.11**	**0.15**	0.39	**1.71**	**0.78**	0.08	1.02	0.03	0.85
	**ICMT**	**-1.17**	**-0.22**	**0.01**	1.01	0.01	0.94	1.04	0.06	0.69	**1.25**	**0.32**	0.41	1.06	0.09	0.42	1.02	0.03	0.74
**Prenylcysteine processing enzymes**	**RCE1**													-1.05	-0.07	0.52	-1.02	-0.02	0.80
	**ZMPSTE24**	1.03	0.04	0.65	-1.06	-0.09	0.16	1.04	0.06	0.58	1.07	0.09	0.35	**1.20**	**0.27**	0.17	**1.20**	**0.27**	0.09
	**RHOA**	-1.02	-0.03	0.77	-1.05	-0.07	0.38	-1.02	-0.03	0.80	**1.15**	**0.20**	0.33	**1.41**	**0.49**	**0.04**	-1.01	-0.02	0.89
	**RHOB**	**-1.15**	**-0.21**	0.15	**-1.33**	**-0.41**	**0.002**	**-1.14**	**-0.19**	0.18	**-1.21**	**-0.28**	0.10	**1.22**	**0.29**	**0.02**	**-1.18**	**-0.23**	**0.01**
	**RHOC**													**1.24**	**0.31**	**0.03**	**-1.15**	**-0.21**	0.08
**GTPases**	**CDC42**	1.05	0.06	0.69	**1.18**	**0.23**	**0.03**	**-1.16**	**-0.22**	0.29	1.03	0.04	0.76	**1.19**	**0.25**	0.29	1.01	0.01	0.92
	**RAC1**	**1.10**	**0.13**	0.07	1.00	0.00	0.95	1.01	0.02	0.82	1.02	0.03	0.66	**1.84**	**0.88**	0.06	**-1.11**	**-0.15**	0.32
	**RAC2**													**-1.10**	**-0.13**	0.21	-1.06	-0.09	0.51
	**KRAS**	**-1.10**	**-0.14**	0.25	-1.05	-0.07	0.58	**1.31**	**0.38**	**0.01**	-1.04	-0.05	0.61	**1.65**	**0.73**	**0.01**	1.09	**0.13**	0.38
	**RAP1A**	**-1.22**	**-0.29**	0.06	**-1.13**	**-0.18**	0.16	-1.04	-0.06	0.73	-1.04	-0.06	0.75	**1.24**	**0.32**	0.16	1.01	0.01	0.91

Values in bold type indicate fold change (FC) ≥ ± 1.10 or $p < 0.05$
